# Label-free metabolic biomarkers for assessing valve interstitial cell calcific progression

**DOI:** 10.1038/s41598-020-66960-4

**Published:** 2020-06-25

**Authors:** Ishita Tandon, Olivia I. Kolenc, Delaney Cross, Isaac Vargas, Shelby Johns, Kyle P. Quinn, Kartik Balachandran

**Affiliations:** Department of Biomedical Engineering, University of Arkansas, Fayetteville, AR 72701 USA

**Keywords:** Optical imaging, Valvular disease

## Abstract

Calcific aortic valve disease (CAVD) is the most common form of valve disease where the only available treatment strategy is surgical valve replacement. Technologies for the early detection of CAVD would benefit the development of prevention, mitigation and alternate therapeutic strategies. Two-photon excited fluorescence (TPEF) microscopy is a label-free, non-destructive imaging technique that has been shown to correlate with multiple markers for cellular differentiation and phenotypic changes in cancer and wound healing. Here we show how specific TPEF markers, namely, the optical redox ratio and mitochondrial fractal dimension, correlate with structural, functional and phenotypic changes occurring in the aortic valve interstitial cells (VICs) during osteogenic differentiation. The optical redox ratio, and fractal dimension of mitochondria were assessed and correlated with gene expression and nuclear morphology of VICs. The optical redox ratio decreased for VICs during early osteogenic differentiation and correlated with biological markers for CAVD progression. Fractal dimension correlated with structural and osteogenic markers as well as measures of nuclear morphology. Our study suggests that TPEF imaging markers, specifically the optical redox ratio and mitochondrial fractal dimension, can be potentially used as a tool for assessing early CAVD progression *in vitro*.

## Introduction

Calcific aortic stenosis or calcific aortic valve disease (CAVD) is a progressive disease involving multiple signaling pathways, endothelial dysfunction, cytokine infiltration, collagen remodeling, as well as lipid and calcium deposition^1–4^. The symptoms and markers for CAVD manifest via both degenerative (apoptotic) and active (osteogenic) mechanisms^5,6^. Aortic valve endothelial and interstitial cells differentiate into an osteoblast-like phenotype, the extracellular matrix becomes thicker and stiffer and calcium mineralization occurs throughout the tissue^7,8^. Aortic stenosis and sclerosis have an increased prevalence in the elderly and contribute to a 50% elevated risk of infarction and other potentially fatal cardiovascular pathologies^2,9,10^. Currently, valve replacement is the preferred treatment method, as other strategies, such as the retardation of calcific progression, prevention and early diagnosis, are non-existent^11^. Diagnostic techniques like echocardiography, cardiac MRI and cardiac CT are widely used, but are only sensitive during later stages of the disease once there is tissue mineralization, and hemodynamic and geometric impairment^12,13^.

Aortic valve interstitial cells (VICs) are the primary cells in the heart valves and are involved in tissue maintenance, repair and remodeling^14^. VICs exist in a quiescent state under healthy conditions and are activated due to injury or disease^15^, potentially differentiating into an osteogenic-like phenotype to potentiate calcification^2^. Current *in vitro* biochemical techniques to assess CAVD are typically destructive as they involve cell lysis or fixation and do not facilitate the longitudinal assessment of CAVD progression over time. That is, there is a dearth of nondestructive, label-free mechanisms to study the structural functional and phenotypic changes occurring in VICs during CAVD progression. Gaining deeper insights into the optical metabolic changes in VICs during disease pathogenesis would thus aid in the development of potential non-invasive tools to track CAVD progression *in vitro*.

Nicotinamide adenine dinucleotide (NADH) and flavin adenine dinucleotide (FAD) are metabolic cofactors that play a key role in carrying electrons from the tricarboxylic acid cycle (TCA cycle) to the electron transport chain in mitochondria. These two molecules exhibit natural autofluorescence^16,17^, which can be detected and quantified using two-photon excited fluorescence (TPEF) microscopy to determine cellular metabolic state through an optical redox ratio (ORR) of FAD/(NADH + FAD)^17–19^. NADH autofluorescence can also be used to assess the mitochondrial organization by determining mitochondrial fractal dimension (FD)^18,20^. Previously, TPEF was used to monitor the progression of osteogenic differentiation in human mesenchymal stem cells (MSCs)^18,21,22^ and ORR and mitochondrial FD was shown to correlate with the osteogenic differentiation of human MSCs^18^. Recently, TPEF imaging was utilized *in vitro* and on *ex vivo* tissue explants to detect mineralization, a key hallmark of CAVD^23^. In the context of VICs, we have previously shown that ORR correlated with cellular proliferative potential when VICs were cultured under different media conditions^24^. We have also previously reported that an increase in pathological stretch reduced the ORR in VICs, suggesting that the involved signaling pathways and VIC pathological function are closely linked to the overall cellular metabolic state^14,19,24^. However, TPEF imaging metrics - ORR and mitochondrial clustering have not yet been shown to predict or correlate with the pathological changes in VICs during early CAVD progression.

The objective of this study was thus to investigate the potential of ORR and mitochondrial organization as label-free markers for tracking early CAVD progression. We seeded porcine aortic VICs as monolayers in quiescent versus osteogenic media on two dimensional soft or stiff substrates. We examined these samples using TPEF microscopy to quantify ORR and mitochondrial FD and simultaneously characterized the CAVD progression in our model using traditional end-point biomarkers, such as calcific nodule quantification, gene expression, cell proliferation and apoptosis. We then correlated ORR and FD with the aforementioned VIC structural and biological metrics. Our results showed that TPEF metrics correlated with the early markers of CAVD progression and thus suggest that TPEF microscopy can be utilized as a label-free non-destructive tool for assessing CAVD progression *in vitro*.

## Results

### Osteogenic cultures on compliant and stiff substrates developed calcific nodules over time

**Figure 1:**
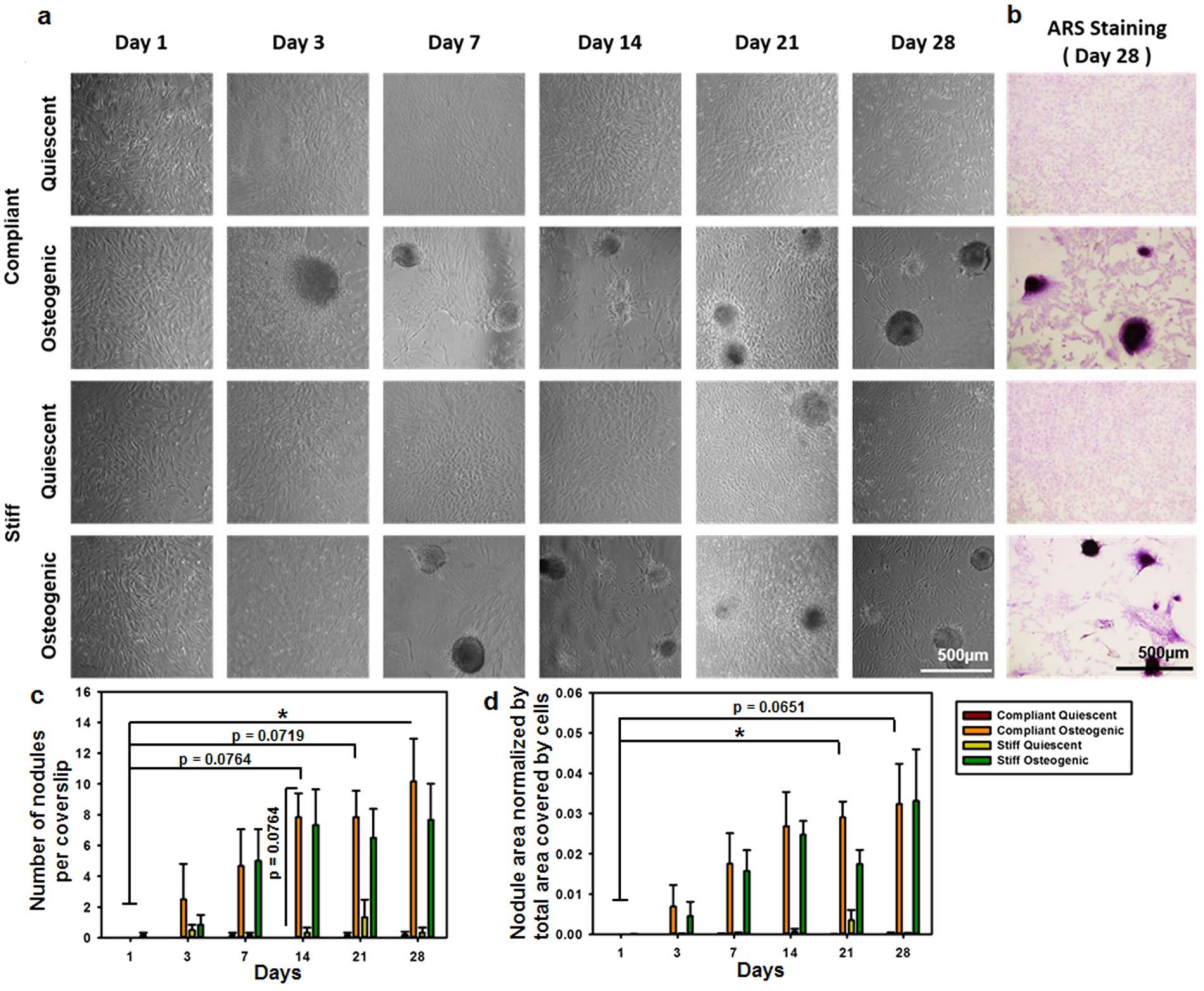
Development of calcific nodules in osteogenic cultures. (**a**) Phase contrast images of VIC cultures for days 1 through 28 under quiescent or osteogenic conditions on compliant or stiff substrates. (**b**) Alizarin Red S stained 28-day VICs under quiescent or osteogenic conditions on compliant or stiff substrates. (**c**) Average number of nodules per coverslip. (**d**) Area covered by the nodules per coverslip normalized by the total area covered by the cells. N = 5–6, *p < 0.05, Scale bar = 500 µm.

Progression of nodule size and number has been used as a classic marker for CAVD progression *in vitro*^2,9^. In our study, VICs cultured under quiescent conditions showed little to no calcium-rich nodules (Fig. 1). VICs under osteogenic conditions developed nodules over time (Fig. 1a), suggesting robust disease progression in our model. Positive Alizarin Red S (ARS) staining on day 28 confirmed that the nodules present in the osteogenic cultures by day 28 were calcific in nature (Fig. 1b). The number of nodules per sample increased with time (Fig. 1c). The number of nodules in compliant substrate osteogenic cultures at day 28 (p = 0.0356) were higher than the number of nodules in quiescent cultures and compliant substrate osteogenic culture at day 1. The normalized area of nodules increased with time (Fig. 1d), with the area of nodules in compliant substrate osteogenic cultures at day 21 (p = 0.0316) being higher than the area of nodules in quiescent cultures and compliant substrate osteogenic culture at day 1.

### Optical Redox Ratio (ORR) decreased at day 14 and then increased again at day 28

**Figure 2:**
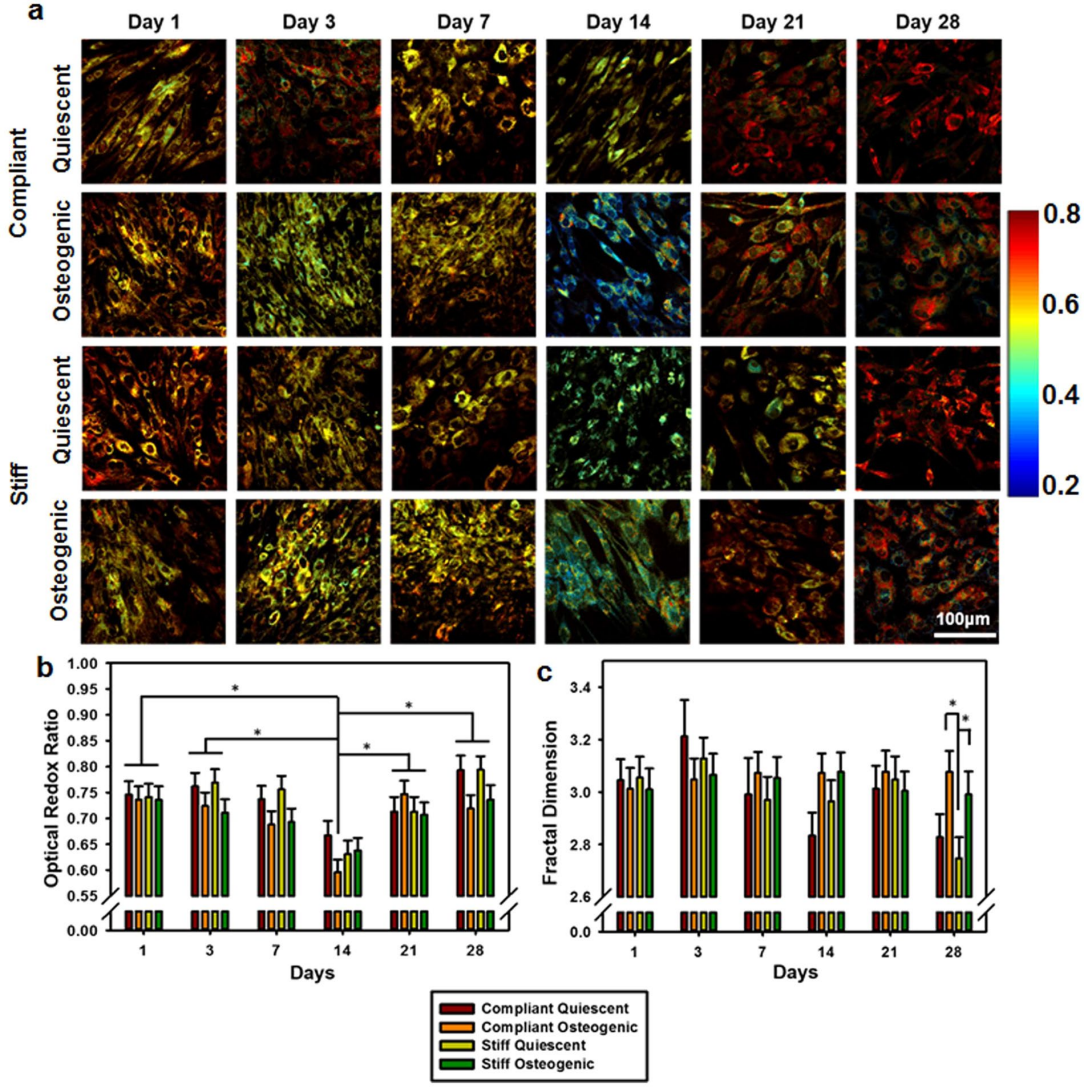
Optical redox ratio and mitochondrial fractal dimension change with osteogenic differentiation of VICs. (**a**) Redox ratio maps of VIC cultures for days 1–28 under quiescent or osteogenic conditions on compliant or stiff substrates. (**b**) Average optical redox ratios and (**c**) Average fractal dimension for days 1 through 28 under quiescent or osteogenic conditions on compliant or stiff substrates. Scale bar = 100 µm. N = 5–6, *p < 0.05.

Label-free TPEF microscopy was performed on live VICs to generate ORR maps (Fig. 2a). Average ORR decreased over time till day 14 and then increased again by day 28, resulting in a lower ORR at day 14 (p < 0.0001) compared to all other time points (Fig. 2b). Overall, ORR for osteogenic cultures was significantly lower than quiescent cultures (p = 0.0072). VICs cultured on compliant substrates showed more pronounced changes in ORR as compared to those cultured on stiff substrates. Within samples cultured on compliant substrates, ORR observed in osteogenic VICs at day 14 was significantly lower than that of VICs at day 1 (p < 0.0001), day 3 (p < 0.001), day 21 (p < 0.005) and day 28 (p < 0.001). Within samples cultured on stiff substrates, ORR osteogenic VICs at day 14 was significantly lower than that observed at day 1 (p = 0.0109), and increased significantly by day 28 (p = 0.0453). Fractal dimension (FD), a metric inversely proportional to mitochondrial clustering, was assessed from the NADH images (Fig. 2c). FD for osteogenic VICs on compliant (p = 0.002) and stiff (p = 0.0267) substrates was significantly higher than that of quiescent VICs on stiff substrate on day 28.

### ORR correlated with VIC osteogenic markers and FD correlated with VIC structural markers

**Figure 3:**
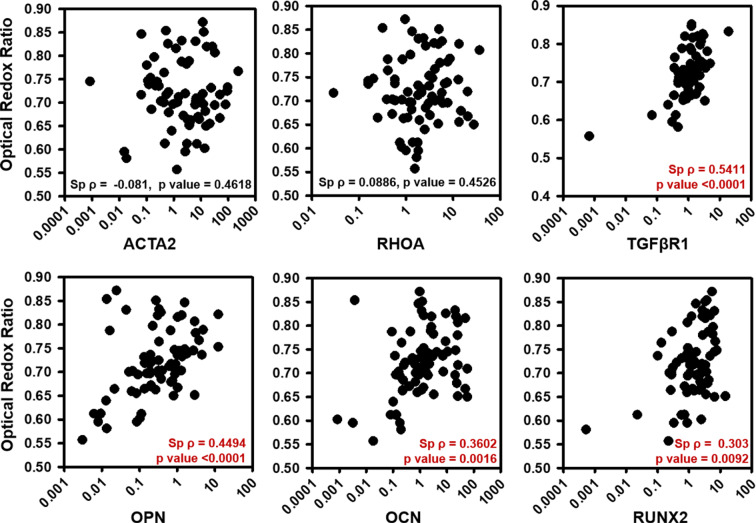
Optical redox ratio correlated with osteogenic markers. Optical redox ratio correlated with (**a**) ACTA2, (**b**) RHOA, (**c**) TGFβR1, (**d**) OPN, (**e**) OCN and (**f)** RUNX2 fold change for VIC cultures for days 1 through 28 under quiescent or osteogenic conditions on compliant or stiff substrates. Spearman’s Rank correlation coefficient is denoted as (Sp ρ).

**Figure 4:**
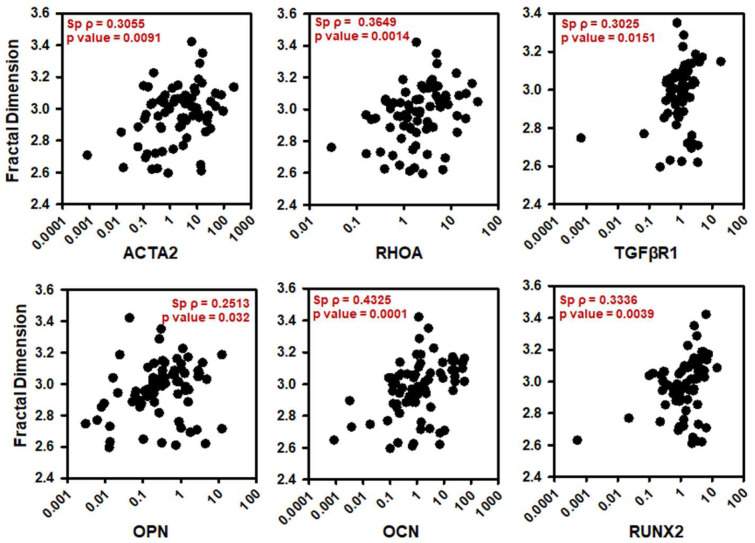
Mitochondrial fractal dimension correlated with structural and osteogenic markers. Fractal Dimension correlated with (**a**) ACTA2, (**b**) RHOA, (**c**) TGFβR1, (**d**) OPN, (**e**) OCN and (**f**) RUNX2 fold change for VIC cultures for days 1 through 28 under quiescent or osteogenic conditions on compliant or stiff substrates. Spearman’s Rank correlation coefficient is denoted as (Sp ρ).

To assess the utility of TPEF markers for tracking CAVD progression *in vitro*, we correlated ORR (Fig. 3) and FD (Fig. 4) with fold change in gene expression of structural, functional and phenotypic markers using Spearman’s rank correlation coefficients. These markers included the genes ACTA2, RHOA, TGFβR1, OPN, OCN and RUNX2. Correlations were performed with respect to early (days 1–7) and late (days 14–28) culture time points, media (quiescent vs osteogenic), substrate (compliant vs. stiff) and their combinations. While ORR correlated mostly with phenotypic and functional markers TGFβR1, OPN, OCN and RUNX2 (Table 1a), FD also correlated with structural markers ACTA2 and RHOA (Table 1b). TGFβR1 codes for transforming growth factor beta (TGFβ) receptor 1, which is upregulated in cells on stiffer matrices and has been shown to steer nodule formation on stiffer matrices via apoptosis instead of osteogenesis^9,25,26^. For all time points and conditions, ORR correlated with TGFβR1 (Sp ρ = 0.5411, p < 0.0001) and these correlations were found to be stronger in stiffer matrices especially in the osteogenic conditions and at later time points (Table 1a). Osteopontin (OPN), a known early biomarker for osteogenic differentiation in VICs^27^, correlated with ORR (Sp ρ = 0.4494, p < 0.0001) for all conditions and time points but showed the strongest correlations at in early osteogenic VICs (Sp ρ = 0.8316, p < 0.0001) (Table 1a). Additionally, RUNX2 is a key transcription factor shown to promote CAVD via multiple effectors^28,29^ and its expression also induces expression of other osteogenic markers like (OPN) and osteocalcin (OCN)^2^. ORR also correlated with TGFβR1 (Sp ρ = 0.609, p = 0.0044), OPN (Sp ρ = 0.8316, p < 0.0001), OCN (Sp ρ = 0.8203, p = 0.0003) and RUNX2 (Sp ρ = 0.5398, p = 0.014) gene expression in early osteogenic VICs. Though RUNX2 had stronger correlation with ORR on stiffer substrates while not showing any correlations on compliant substrates, OCN correlated with ORR much stronger on compliant substrates especially in the osteogenic conditions (Table 1a). Finally, the structural markers ACTA2 and RHOA play important roles in cellular organization. ACTA2 encodes alpha smooth muscle actin (αSMA) responsible for generation of stress fibers, and RHOA encodes a molecular switch involved in actin re-organization during nodule formation^30,31^. TGFβ1 is known to also induce αSMA expression and myofibroblastic differentiation in VICs, which increases with an increase in substrate stiffness^32^. FD correlated with TGFβR1 (Sp ρ = 0.4062, p = 0.0076), OPN (Sp ρ = 0.4803, p = 0.0015), OCN (Sp ρ = 0.4393, p = 0.0036) and RUNX2 (Sp ρ = 0.4611, p = 0.0021) gene expression for days 1–7 and with ACTA2 (Sp ρ = 0.4616, p = 0.0077), RHOA (Sp ρ = 0.03578, p = 0.0102) and OCN (Sp ρ = 0.4568, p = 0.0086) gene expression for days 14–28 for all groups (Table 1b). FD showed stronger correlations with RHOA in early timepoints especially in early osteogenic VICs (Sp ρ = 0.5218, p = 0.0183). FD showed strong correlation with ACTA2 in later time points especially in stiffer matrices (Sp ρ = 0.5588, p = 0.0197), with FD correlating strongly and with TGFβR1 (Sp ρ = 0.502, p = 0.0173) in early VICs especially on stiffer matrices (Table 1b).

**Table 1:** Correlation of ORR with gene expression.

		ORR					
		ACTA2	RhoA	TGFβR1	OPN	OCN	Runx2
**a**							
All	**Sp ρ**	−0.081	0.0886	**0.5411**	0.4494	0.3602	0.303
	**p - value**	0.4618	0.4526	**<0.0001**	<0.0001	0.0016	0.0092
Early (Day 1–7)	**Sp ρ**	−0.0104	0.1237	0.4642	**0.6557**	0.4015	0.3507
	**p - value**	0.9492	0.4353	0.002	**<0.0001**	0.0084	0.0228
Late (Day 14–21)	**Sp ρ**	−0.1063	0.0671	**0.6567**	0.3078	0.3606	0.3031
	**p - value**	0.5625	0.7153	**0.0009**	0.0866	0.0426	0.0974
Quiescent	**Sp ρ**	−0.2176	−0.0432	0.429	0.4093	0.2648	0.2237
	**p - value**	0.2164	0.8033	0.018	0.0146	0.1186	0.1964
Osteogenic	**Sp ρ**	−0.128	0.2343	**0.5838**	0.448	0.4889	0.3856
	**p - value**	0.9392	0.1569	**0.0003**	0.0048	0.0018	0.0168
Early Osteogenic	**Sp ρ**	−0.0421	0.3338	**0.609**	**0.8316**	**0.7203**	**0.5398**
	**p - value**	0.8601	0.1503	**0.0044**	**<0.0001**	**0.0003**	**0.014**
Late Osteogenic	**Sp ρ**	0.2136	0.2652	**0.6**	0.1973	0.3709	0.3481
	**p - value**	0.3947	0.2875	**0.0233**	0.4326	0.1297	0.1569
Stiff	**Sp ρ**	−0.0813	0.0417	**0.5997**	0.4014	0.3263	0.3627
	**p - value**	0.6275	0.801	**0.0002**	0.0125	0.0427	0.0233
Compliant	**Sp ρ**	−0.0955	0.1252	0.4323	0.4691	0.404	0.1771
	**p - value**	0.5911	0.4736	0.0171	0.0045	0.0161	0.3163
Stiff Osteogenic	**Sp ρ**	−0.0421	0.0614	**0.6373**	0.4513	0.4776	0.4284
	**p - value**	0.8641	0.8028	**0.0059**	0.0525	0.0386	0.0672
Compliant Osteogenic	**Sp ρ**	0.0333	0.4281	**0.5294**	0.3749	0.4996	0.3468
	**p - value**	0.8922	0.0675	**0.0289**	0.1138	0.0294	0.1485
Early Stiff	**Sp ρ**	−0.0078	0.153	**0.6115**	**0.6091**	0.3958	0.4037
	**p - value**	0.9733	0.4966	**0.0025**	**0.0034**	0.0682	0.0624
Early Compliant	**Sp ρ**	−0.0404	0.0451	0.215	**0.6436**	0.4211	0.2586
	**p - value**	0.8697	0.8502	0.3626	**0.0022**	0.0645	0.2709
Late Stiff	**Sp ρ**	−0.0784	−0.0931	**0.6294**	0.2918	0.3262	0.3826
	**p - value**	0.7684	0.7222	**0.0283**	0.2557	0.2013	0.1296
Late Compliant	**Sp ρ**	−0.1607	0.3	**0.7333**	0.3378	0.4486	0.1232
	**p - value**	0.5672	0.2773	**0.0158**	0.2182	0.0935	0.6747
Moderate correlation (R > 0.3)							
**High correlation (R** > **0.5)**							
		**FD**					
		**ACTA2**	**RhoA**	**TGFβR1**	**OPN**	**OCN**	**Runx2**
**b Correlation of FD with gene expression**.							
All	**Sp ρ**	0.3055	0.3649	0.3025	0.2513	0.4325	0.3336
	**p - value**	0.0091	0.0014	0.0151	0.032	0.0001	0.0039
Early (Day 1–7)	**Sp ρ**	0.1807	0.3921	0.4062	0.4803	0.4393	0.4611
	**p - value**	0.2646	0.0102	0.0076	0.0015	0.0036	0.0021
Late (Day 14–21)	**Sp ρ**	0.4626	0.3578	0.2117	0.0748	0.4568	0.2686
	**p - value**	0.0077	0.0444	0.3442	0.6841	0.0086	0.144
Quiescent	**Sp ρ**	0.2663	0.1797	0.1221	0.2026	0.2694	0.1385
	**p - value**	0.1279	0.2944	0.5203	0.2347	0.1121	0.4274
Osteogenic	**Sp ρ**	0.2934	0.4667	0.4946	0.339	0.4928	0.4782
	**p - value**	0.0739	0.0031	0.0029	0.0374	0.0017	0.0024
Early Osteogenic	**Sp ρ**	0.3805	**0.5218**	0.388	0.3789	0.4902	0.4075
	**p - value**	0.098	**0.0183**	0.091	0.0094	0.0951	0.70745
Late Osteogenic	**Sp ρ**	0.2425	0.3911	**0.5868**	0.3182	0.4773	0.3905
	**p - value**	0.3322	0.1085	**0.0274**	0.1982	0.0452	0.1091
Stiff	**Sp ρ**	0.4037	0.3617	0.2388	0.1334	0.3751	0.285
	**p - value**	0.012	0.0236	0.1738	0.4246	0.0186	0.0787
Compliant	**Sp ρ**	0.1756	0.3291	0.329	0.3551	0.4628	0.3837
	**p - value**	0.3207	0.0535	0.0758	0.0363	0.0051	0.0251
Stiff Osteogenic	**Sp ρ**	0.4596	0.4649	**0.5221**	0.2511	**0.5127**	**0.5338**
	**p - value**	0.0477	0.0449	**0.0316**	0.2998	**0.0248**	**0.0186**
Compliant Osteogenic	**Sp ρ**	0.207	0.4351	0.3824	0.403	0.4083	0.4346
	**p - value**	0.3951	0.0626	0.1299	0.0871	0.0827	0.063
Early Stiff	**Sp ρ**	0.239	0.4534	**0.502**	**0.5156**	0.4331	**0.5381**
	**p - value**	0.2968	0.0341	**0.0173**	**0.0167**	0.0441	**0.0098**
Early Compliant	**Sp ρ**	0.1281	0.3609	0.3203	0.4827	0.4977	0.391
	**p - value**	0.6013	0.118	0.1686	0.0311	0.0255	0.0883
Late Stiff	**Sp ρ**	**0.5588**	0.2868	−0.042	−0.1533	0.3814	0.2759
	**p - value**	**0.0197**	0.2644	0.897	0.557	0.1309	0.2838
Late Compliant	**Sp ρ**	0.2929	0.35	0.4545	0.3128	**0.5165**	0.3564
	**p - value**	0.2895	0.2009	0.1869	0.2563	**0.0487**	0.211
Moderate correlation (R > 0.3)							
**High correlation (R** > **0.5)**							

### Nuclear area and aspect ratio correlated with fractal dimension

**Figure 5:**
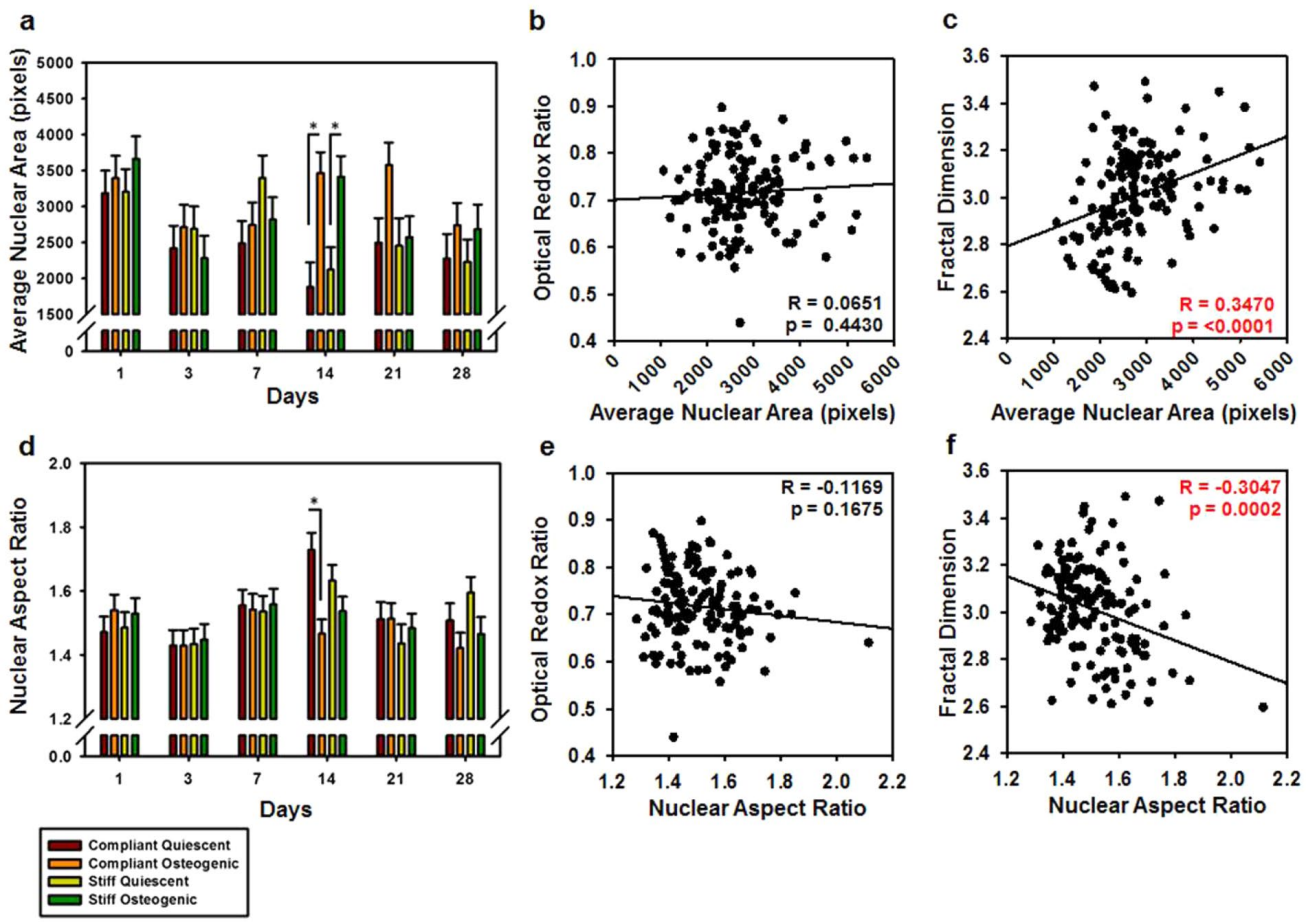
Nuclear morphology changes correlated with mitochondrial fractal dimension during VIC osteogenic differentiation. (**a**) Average nuclear area in pixels for VIC cultures for days 1 through 28 under quiescent or osteogenic conditions on compliant or stiff substrates. (**b**) Optical redox ratio correlated with average nuclear area. (**c**) Fractal dimension correlated with the average nuclear area. (**d**) Nuclear aspect ratio for VIC cultures for days 1 through 28 under quiescent or osteogenic conditions on compliant or stiff substrates. (**e**) Optical redox ratio correlated with nuclear aspect ratio. (**f**) Fractal dimension correlated with the nuclear aspect ratio. N = 5–6, *p < 0.05. Pearson’s correlation coefficient is denoted as (R).

We have previously demonstrated that VIC shape influences its functional characteristics such as contractility, metabolism and proliferation^19,33^. We have also shown that VIC aspect ratio under normal and osteogenic culture conditions correlates with nuclear aspect ratio^33^. In the current study, ORR maps were utilized to assess nuclear area (Fig. 5a) and nuclear aspect ratio (AR) (Fig. 5d) and then correlated with ORR and FD. Nuclear area was significantly lower at days 3, 7, 14, 21 and 28 as compared to day 1 (p < 0.001). Nuclear area was significantly higher for osteogenic VICs as compared to quiescent VICs on both compliant substrates (p = 0.0008) and stiff substrates (p = 0.0017) at day 14, which also interestingly coincided with the lowest ORR. Overall, nuclear area was significantly higher for osteogenic VICs (p = 0.0031) compared to the quiescent VICs, especially on compliant substrates (p = 0.0301). AR decreased significantly at day 3, as compared to day 1 (p = 0.0364) and then increased again at day 7 (p < 0.0001) and day 14 (p < 0.0001). AR decreased at day 21 (p = 0.0062) and day 28 (p = 0.0235), as compared to day 14. At the 14-day time point, osteogenic VICs on complaint substrates had significantly lower AR as compared to quiescent VICs on complaint substrate (p = 0.05). When correlating nuclear area with ORR and FD, we observed that ORR did not correlate with nuclear area (Fig. 5b), but FD positively correlated with nuclear area (R = 0.3470, p < 0.0001) (Fig. 5c). The nuclear AR was then correlated with ORR and FD. ORR did not correlate with nuclear AR (Fig. 5e), however, FD negatively correlated with nuclear AR (R = −0.3047, p = 0.0002) (Fig. 5f).

### Nodules in osteogenic cultures were apoptotic and proliferative in nature

**Figure 6:**
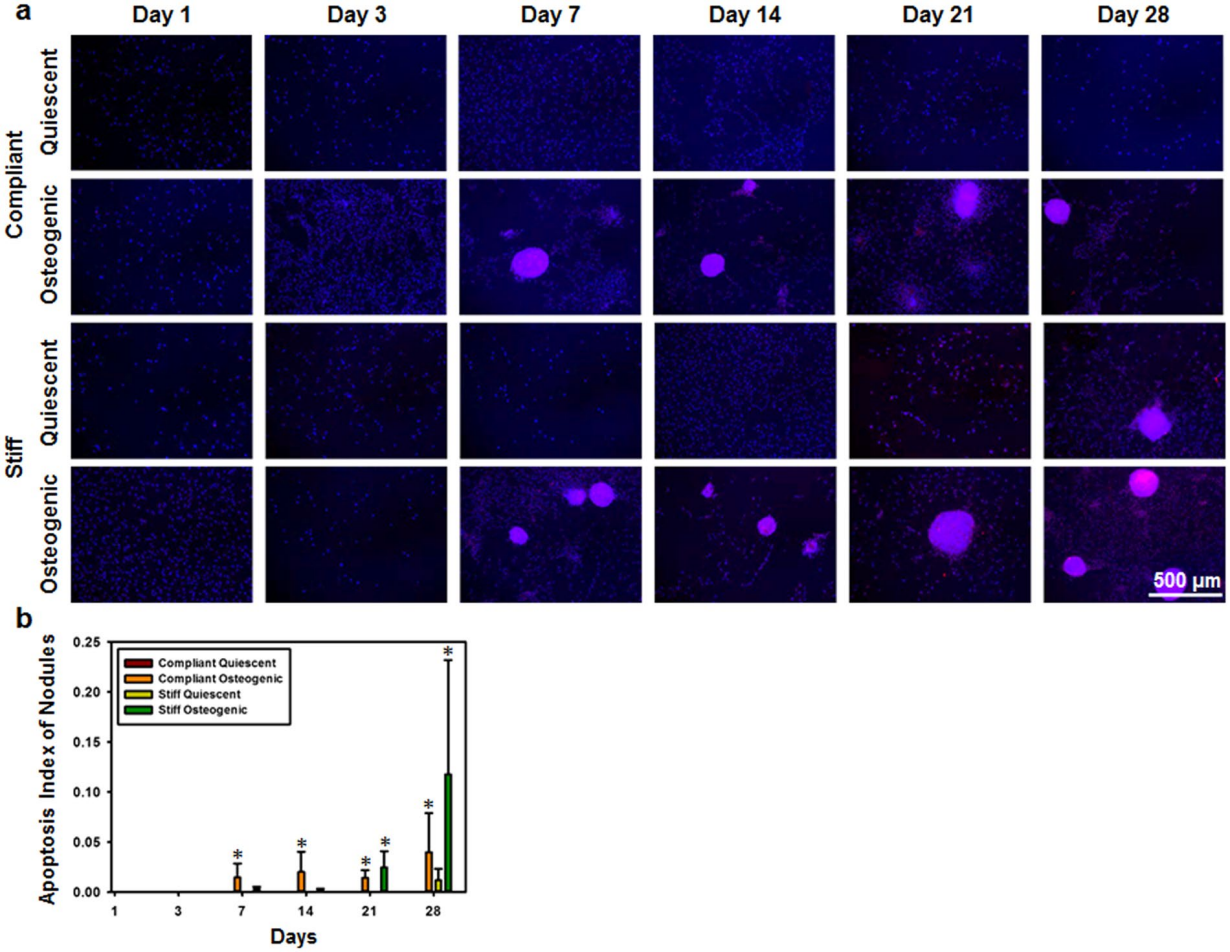
Calcific nodules in osteogenic cultures were apoptotic in nature. (**a**) Representative Caspase immunostains (Caspase in red and nuclei in blue) of VIC cultures for days 1 through 28 under quiescent or osteogenic conditions on compliant or stiff substrates. (**b**) Average apoptosis index of nodules for days 1 through 28 under quiescent or osteogenic conditions on compliant or stiff substrates. N = 3, Scale bar = 500 µm. *p < 0.05 compared to corresponding quiescent media condition.

**Figure 7:**
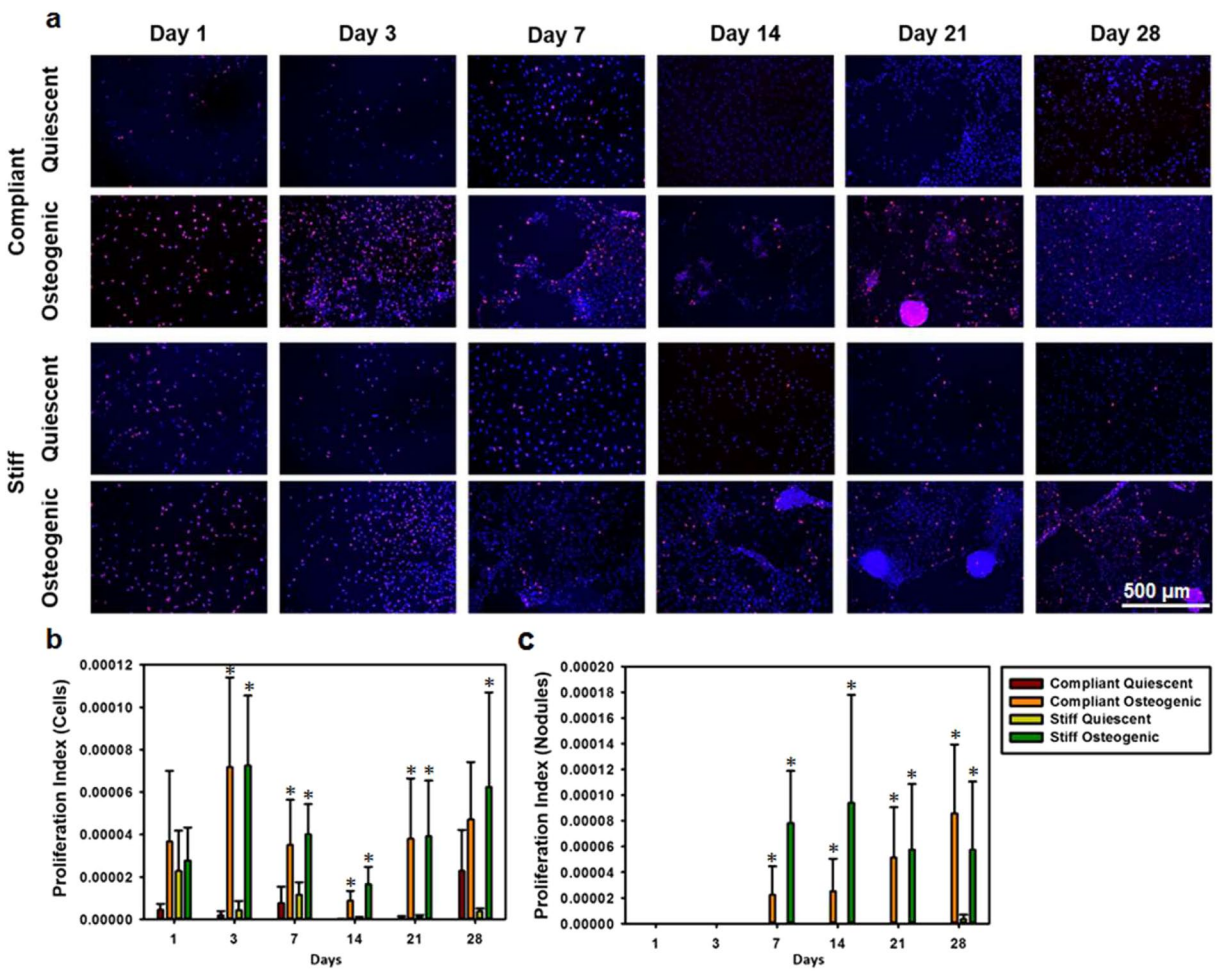
Cells and nodules in osteogenic cultures were proliferative in nature. (**a**) Representative Ki67 immunostains (Ki67 in red and nuclei in blue) of VIC cultures for days 1 through 28 under quiescent or osteogenic conditions on compliant or stiff substrates. Average proliferation index of (**b**) cells and (**c**) nodules for days 1 through 28 under quiescent and osteogenic conditions on compliant or stiff substrates. N = 3, Scale bar = 500 µm. *p < 0.05 compared to corresponding quiescent media condition.

VICs have been known to undergo apoptosis and proliferation during CAVD progression. Additionally, apoptosis is a known marker for dystrophic calcification^34^. Caspase staining revealed the presence of apoptotic cells in the nodules (Fig. 6a). Mean apoptosis index of the nodules was quantified (Fig. 6b) and was found to be significantly higher in the osteogenic media conditions compared to their corresponding quiescent media conditions (p < 0.05) and correlated with RHOA gene expression (R = 0.4049, p = 0.0497). Ki67 staining revealed that the cells were proliferative within, and outside, the nodules (Fig. 7a). Proliferation index for cells outside of the nodules (Fig. 7b) and those within the nodules (Fig. 7c) was quantified and it was seen that the proliferation index was significantly higher in the osteogenic media conditions, compared to their corresponding quiescent media conditions (p < 0.05). The proliferation index for cells outside of the nodules correlated with OCN gene expression (R = 0.5527, p = 0.0051), while the proliferation index for cells within the nodules correlated with RUNX2 gene expression (R = 0.7080, p = 0.0001).

## Discussion

Currently, there are few tools available to non-invasively monitor CAVD progression *in vitro*, thus somewhat limiting the efficient development of therapies for this disease. In this study, we hypothesized that non-invasive TPEF metrics would correlate with the early phenotypic changes of VICs during CAVD progression. This hypothesis was tested using an *in vitro* CAVD model via the modulation of media and substrate stiffness in two-dimensional VIC cultures. The presence of calcific nodules, apoptosis and proliferation of cells within nodules, and gene expression were used to assess functional changes in VICs. Nuclear morphology was used to describe the structural properties of VICs. TPEF metrics of ORR and FD were then correlated with these structural and functional markers.

ORR and FD were previously shown to correlate with the osteogenic differentiation of mesenchymal stem cells^18^. In assessing the ORR of VICs cultured under quiescent and osteogenic conditions, we show for the first time that ORR changed over time during early CAVD progression. Specifically, the ORR was significantly lower at day 14 and then increased again at day 21. This trend was similar to that observed during osteogenic differentiation of mesenchymal stem cells *in vitro*^18^. In our model, more pronounced changes were observed in the ORR compared to mitochondrial reorganization as measured by fractal dimension (FD). It has been previously demonstrated that changes in ORR can occur before changes in mitochondrial organization within the cell^18^, and our results support this concept as well. Additionally, we also reported a correlation between osteogenic gene expression and ORR and FD as seen in prior studies^18^, suggesting the possibility of using TPEF metrics to predict the CAVD disease process.

Digging deeper into our results, we observed that TGFβR1 expression significantly correlated with ORR, which was expected given that TGFβ1 signaling has a major role in inducing disease during valve calcification. RUNX2 expression is known to predict the early osteogenic lineage of the cell^25^, and thus correlated well with ORR during early timepoints. Additionally, RUNX2 and OCN correlating with ORR based on stiffness, further supported the mechanosensitive nature of RUNX2 signaling in VICs. RUNX2 and OCN also correlated with VIC proliferation index, suggesting that osteogenic cells tended to be more proliferative. In the context of the above results, it is also important to note that while our quiescent media and osteogenic media formulations contain differing amounts of FBS, previous reports have rigorously characterized these specific media formulations to maintain quiescent and osteogenic cells, respectively^15,24,25,33–39^. Our results also agree with those accounted previously that demonstrated that quiescent VICs are less proliferative than VICs in 10% FBS containing osteogenic media^25,36,38–40^. Future studies will focus on the effect of individual media formulations and FBS concentrations on TPEF metrics and cell proliferation.

OPN expression has been known to be an early biomarker for CAVD and has been used as a serum marker to study the progression of the disease when it is asymptomatic^27,41^. In our study, OPN and OCN correlated with the ORR, specifically for the early timepoints. While TGFβR1, RUNX2, OPN, and OCN gene expression all were highly correlated with ORR for early timepoints, OPN expression showed the strongest correlation coefficient. OPN did not show any correlations to ORR in late time points, but correlated well with osteogenic cultures under both stiff and compliant cultures at early timepoints. Taken as a whole, our results suggest that the correlation of ORR with OPN can potentially be exploited as a predictor of early metabolic changes during CAVD progression and serve as a biomarker for early disease^27,41^.

Alpha SMA organizes into stress fibers and affects stiffness, force generation, and cell shape^30^. We have previously shown that elongated cells had higher expression of proliferation and αSMA expression^19^. Dystrophic calcification has been known to be associated with apoptosis and prolonged αSMA activation in VICs^30^. In our study, ACTA2 did not correlate with ORR but significantly correlated with mitochondrial FD. Stronger correlations were observed between ACTA2 and FD in osteogenic VICs on stiffer substrates, especially at later time points. This suggests that the role played by ACTA2 in stress fiber formation may also affect the regulation of the arrangement of mitochondria in adjusting to the energy demands of the cell under altered stiffness and osteogenesis. Stiffness dependent correlations of TGFβR1 with FD at early timepoints may suggest its role in triggering the aforementioned ACTA2 mediated mitochondrial arrangement^32^. Additionally, the RHOA gene, which codes for a mechanotransductive protein that regulates the formation of nodules^42^, also correlated with nodule apoptotic index. RHOA has been known to be activated and expressed at higher levels in stiffer matrices and promote the upregulation of RUNX2^43^. Like ACTA2, RHOA did not correlate with ORR but significantly correlated with mitochondrial FD. RHOA has been shown to increase ACTA2 expression, ultimately increasing stress fiber formation^30,31^. This sequence of events is in line with our results that showed that RHOA gene expression correlated with FD at earlier time points, whereas ACTA2 gene expression correlated with FD for later time points.

Due to the impact of VIC shape on cellular function, changes in nuclear area and aspect ratio were measured to provide context for the differences we observed in mitochondrial FD. These measures significantly correlated with mitochondrial FD, with nuclear area positively correlated with FD, and aspect ratio negatively correlated with FD. This suggests that mitochondrial clustering decreased with an increase in nuclear area and a decrease in nuclear aspect ratio. We observed an increase in nuclear area and FD in osteogenic VICs, suggesting these cells had decreased mitochondrial clustering. Similar to our previous study^33^, the VICs in osteogenic conditions tended to have a lower nuclear aspect ratio, reinforcing the idea that osteogenic cells have reduced mitochondrial clustering. Disorganization of mitochondria has previously been associated with other cardiac ailments^44^, but the specific functional role of mitochondrial clustering in valve pathophysiology is yet to be elucidated. It is also not fully understood if the increased area and decreased AR of nuclei in osteogenic VICs is a cause or effect of the disorganization of mitochondria.

Overall, the correlations we observed between nuclear morphology, gene expression and the TPEF metrics of ORR and FD, suggest that VIC phenotype and shape have an effect on the metabolic state of the cell and its mitochondrial arrangements. This study showed that TPEF microscopy and its derived metrics can be used to assess pathophysiological changes in the structure, function and phenotype of VICs undergoing osteogenic differentiation, suggesting that TPEF imaging markers can serve as a label-free, non-destructive tool to study the cellular changes occurring during CAVD progression *in vitro*. Future studies will dig deeper into the exact metabolic changes that occur during CAVD progression and how they relate to observed alteration in measured TPEF metrics, and whether any of these techniques can be translated *in vivo*.

## Materials and Methods

### Valve interstitial cell isolation and seeding

Standard cell culture procedures were followed as published earlier^19,33^. Briefly, porcine hearts were acquired from a local abattoir and aortic valves were utilized to aseptically isolate the valve interstitial cells (VICs). Human fibronectin (Corning, Corning, NY) at a concentration of 100 μg/ml was coated on stiff or compliant PDMS-coated coverslips (Supplementary Table S1), before seeding the cells^33^. VICs were conditioned in quiescent media (Dulbecco’s Modified Eagle Medium, with 50 U/ml penicillin, 50 U/ml streptomycin, and 10 mM HEPES, supplemented with 2% fetal bovine serum (FBS), 10 ng/ml FGF-2 and 50 ng/ml insulin)^36^ for 14 days before seeding onto the coverslips. These quiescent VICs from passages three to seven were then seeded at 500,000 cells/coverslip and cultured in either quiescent or pro-osteogenic (Dulbecco’s Modified Eagle Medium, with 50 U/ml penicillin, 50 U/ml streptomycin, and 10 mM HEPES, supplemented with 10% FBS, 10 mM β-glycerophosphate, 100 nM dexamethasone, and 50 µg/ml ascorbic acid) conditions^33^ for 1, 3, 7, 14, 21, and 28 days before analyses were performed as outlined below.

### Assessment and quantification of calcific nodules

Three to four fields of view (FOV) from five to six biological replicates per time point per condition were imaged using brightfield phase contrast microscopy before they were utilized for other assays at the 1, 3, 7, 14, 21, and 28-day time points. An image editing software, Fiji Image J (National Institutes of Health) was used to manually count and measure the number and area of nodules per field of view. At the 28-day time-point, the samples were fixed in 4% paraformaldehyde (Electron Microscopy Sciences, Hatfield, PA) with 1% Triton X-100 (Electron Microscopy Sciences) and incubated with 40 mM Alizarin Red S (ARS; Sigma-Aldrich, St. Louis, MO) solution for 30 minutes with gentle shaking^45^. The samples were then washed in deionized water and brightfield images were acquired using a Nikon Eclipse (Ci-S) microscope in conjunction with NIS Elements software.

### Two-photon excited fluorescence (TPEF) imaging and analysis

At the aforementioned time points, separate samples were transferred to a heated microincubator chamber (37 °C) and media exchanged with 2 ml Tyrode’s salt solution buffer (Sigma-Aldrich). A Bruker Ultima Investigator laser scanning microscope (Middleton, Wisconsin) inverted for cell culture imaging and equipped with an ultrafast Ti:Sapphire tunable laser source (Mai Tai HP; Spectra-Physics, Santa Clara CA) was used to acquire images via a water immersion objective (20×; NA = 1; 2.5x digital zoom) (Olympus, Tokyo, Japan) and non-descanned GaAsP photomultiplier tubes (PMT) (H10770PB-40, Hamamatsu, Shizuoka, Japan). NADH fluorescence was captured with a 460 (±20) nm band pass filter at 755 nm excitation, and FAD fluorescence with a 525 (±25) nm band pass filter at 860 nm excitation. NADH and FAD autofluorescence intensities were normalized by PMT gain and laser power, with PMT gain normalized to fluorescein concentrations (0.1 µM to 20 µM in Tris Buffer of pH 8) as in previous studies^18,46^. PMT gain and laser power were kept constant and laser power was read after each imaging session^18,19^. A custom MATLAB code was used to generate redox images by pixel-wise computation of fluorescence intensities as FAD/(NADH + FAD) after normalization. Optical redox ratios (ORR) were calculated using averaged and normalized field intensities of FAD and NADH images as FAD/(NADH + FAD). NADH fluorescence intensity maps were utilized to assess the spatial arrangement of mitochondria using a custom MATLAB code. Specifically, a modified blanket method was utilized to obtain the mitochondrial fractal dimension, a metric defining the closeness of mitochondrial arrangement, on a pixel-by-pixel basis^20^. At least two to four fields of view were taken from five to six separate samples per time point, and per treatment group^18,19^.

### Quantitative real-time polymerase chain reaction and correlation analysis

Quantitative real-time polymerase chain reaction (qRT-PCR) was used to quantify gene expression of ACTA2, RHOA, TGFβR1, OPN, OCN, and RUNX2 using previously published methods^33^. Briefly, a QIAGEN RNeasy Plus assay kit (Qiagen, Hilden, Germany) was utilized to isolate and purify total mRNA from VIC samples. The mRNA was then reverse transcribed to cDNA using iScript RT supermix (Bio-Rad, Hercules, CA). QRT-PCR was performed using the SsoAdvanced Universal SYBR Green supermix (Bio-Rad) in a CFX96 real-time system (BIO-RAD) to quantify the relative expression of genes (Table 2) with respect to the house keeping gene 18S (ΔCt). All primers were obtained from ThermoFisher Scientific (Waltham, MA). The average of delta Ct for all 4 groups for day 1 was then used to normalize individual delta Ct values for all groups at all timepoints to obtain the ΔΔCt. Fold change was then calculated as 2^−ΔΔCt^ and graphed. Three to seven samples per time point and per treatment group were utilized for the analysis. The fold change of each sample was correlated with its corresponding ORR and FD.

**Table 2:** Primer Sequences for qRT-PCR.

Target	Gene	Forward Sequence	Reverse Sequence
αSMA^47^	ACTA2	5′-CAGTTTTCCCTTCCATCGTG-3′	5′-TGGGGTATTTCAAGGTCAGG-3′
RhoA^10^	RHOA	5′-ACCAGTTCCCAGAGGTGTATGT-3′	5′-TTGGGACAGAAATGCTTGACTTC-3′
TGFβ Receptor1^26^	TGFβR1	5′- GACGGCATTCCAGTGTTTCT-3’	5′-TGCACATACAAATGGCCTGT-3′
Osteopontin^4^	OPN	5′-TTGCTAAAGCCTGACCCATCT-3′	5′-CGTCGTCCACATCGTCTGTT-3′
Osteocalcin^4^	OCN	5′-TCAACCCCGACTGCGACGAG-3′	5′-TTGGAGCAGCTGGGATGATGG-3′
RUNX2^42^	RUNX2	5′-GCACTACCCAGCCACCTTTA-3′	5′-TATGGAGTGCTGCTGGTCTG-3′
18S^47^		5′-ATTCCGATAACGAACGAGACT-3′	5′-GGACATCTAAGGGCATCACAG-3′

### Nuclear morphology analysis

ORR maps were additionally used to quantify nuclear aspect ratio and area. Five to eight cells per image field were analyzed using Fiji Image J using previously published methods^33^. Average nuclear area and aspect ratio per treatment group per condition were then calculated and correlated to the average ORR and FD for that treatment group.

### Apoptosis and proliferation analysis

Separate samples were fixed using 4% paraformaldehyde (Electron Microscopy Sciences) with 1% Triton X-100 (Electron Microscopy Sciences), blocked with 10% goat serum (Life Technologies, Carlsbad, CA), and were immunolabeled with antibodies against either cleaved caspase 3 (1:400; Cell Signaling Technology, Danvers, MA) to assess apoptosis, or Ki67 (1: 100; Abcam, Cambridge, MA) to assess proliferation. Samples were then fluorescently labelled with appropriate Alexa Fluor-594 conjugated (1:200; life Technologies) secondary antibody and 4′,6-diamidino-2-phenylindole (DAPI, 1:200; Life Technologies) to stain the nuclei. The samples were mounted onto glass coverslips using Prolong Gold antifade agent (Life Technologies) and imaged using a Nikon epifluorescence microscope in conjunction with NIS Elements software. Technical controls were utilized to assess specificity of the secondary antibody (Supplementary figure S4). (Fiji Image J software was utilized to assess the area of positive caspase expression in the nodules using a constant threshold value. An apoptosis index of the nodules was calculated by normalizing the caspase positive area by the total area of nodules per sample. Fiji Image J software was utilized to assess the number of cells with positive Ki67 expression per field of view. The Ki67 positive cells within the nodules were analyzed separately from the outside the nodules. Proliferation index of the nodules was calculated by normalizing the Ki67 positive cells by the total area of nodules per sample. The proliferation index of the cells was calculated by dividing the total number of Ki67 positive cells by the total cell coverage area.

### Statistical analysis

All data was graphically presented as mean ± standard error of means (SEM). Normally distributed data was analyzed using three-way analysis of variance (ANOVA) and Tukey’s post-hoc tests. For the analysis of ORR, FD, nuclear area and aspect ratio, each FOV was nested within the sample and each sample was nested within the stiffness and media condition. Both sample and FOV were considered a random variable. ANOVA on ranks with Dunn’s multiple comparisons test was used for non-parametric data. A comparison was considered significant if its p-value was less than 0.05. Pearson’s correlation coefficient was used for the correlation analysis. Significant correlations were defined based on a null hypothesis of uncorrelated data (R = 0). If the data was non-normal, Spearman’s rank correlation coefficient was used. All the statistical and correlation analyses was performed using JMP and graphing was performed using SigmaPlot (Systat Software Inc) software.

## Supplementary Information

Supplementary Information.

## References

[1] Lerman DA, Prasad S, Alotti N (2015). Calcific Aortic Valve Disease: Molecular Mechanisms and Therapeutic Approaches. European cardiology.

[2] Leopold JA (2012). Cellular mechanisms of aortic valve calcification. Circulation. Cardiovascular interventions.

[3] Mahler, G. J. & Butcher, J. T. Inflammatory Regulation of Valvular Remodeling: The Good(?), the Bad, and the Ugly. *International Journal of Inflammation* 2011, 10.4061/2011/721419 (2011). 10.4061/2011/721419 PMC3139860 21792386

[4] Gomez-Stallons MV, Wirrig-Schwendeman EE, Hassel KR, Conway SJ, Yutzey KE (2016). Bone Morphogenetic Protein Signaling Is Required for Aortic Valve Calcification. Arteriosclerosis, thrombosis, and vascular biology.

[5] Rajamannan NM (2011). Calcific Aortic Valve Disease: Not Simply a Degenerative Process A Review and Agenda for Research from the National Heart and Lung and Blood Institute Aortic Stenosis Working Group. Circulation.

[6] Goldbarg SH, Elmariah S, Miller MA, Fuster V (2007). Insights Into Degenerative Aortic Valve Disease. Journal of the American College of Cardiology.

[7] Wang H, Leinwand LA, Anseth KS (2014). Cardiac valve cells and their microenvironment—insights from *in vitro* studies. Nat Rev Cardiol.

[8] Balachandran K, Sucosky P, Jo H, Yoganathan AP (2010). Elevated Cyclic Stretch Induces Aortic Valve Calcification in a Bone Morphogenic Protein-Dependent Manner. The American Journal of Pathology.

[9] Hutcheson JD, Aikawa E, Merryman WD (2014). Potential drug targets for calcific aortic valve disease. Nat Rev Cardiol.

[10] Lerman, D. A., Prasad, S. & Alotti, N. Using Na3PO4 to Enhance *In vitro* Animal Models of Aortic Valve Calcification. *Int J Cardiovasc Res* **5** (2016). 10.4172/2324-8602.1000250 PMC4930138 27376093

[11] Beckmann E, Grau JB, Sainger R, Poggio P, Ferrari G (2010). Insights into the use of biomarkers in calcific aortic valve disease. The Journal of heart valve disease.

[12] Lindman BR, Bonow RO, Otto CM (2013). Current Management of Calcific Aortic Stenosis. Circ Res.

[13] Kanwar A, Thaden JJ, Nkomo VT (2018). Management of Patients With Aortic Valve Stenosis. Mayo Clin Proc.

[14] Kamel PI (2014). Metabolic regulation of collagen gel contraction by porcine aortic valvular interstitial cells. J R Soc Interface.

[15] Liu AC, Joag VR, Gotlieb AI (2007). The Emerging Role of Valve Interstitial Cell Phenotypes in Regulating Heart Valve Pathobiology. The American Journal of Pathology.

[16] Bartolome F, Abramov AY (2015). Measurement of mitochondrial NADH and FAD autofluorescence in live cells. Methods Mol Biol.

[17] Kolenc OI, Quinn KP (2017). Evaluating Cell Metabolism Through Autofluorescence Imaging of NAD(P)H and FAD. Antioxidants & Redox Signaling.

[18] Quinn KP (2013). Quantitative metabolic imaging using endogenous fluorescence to detect stem cell differentiation. Sci Rep.

[19] Lam NT, Muldoon TJ, Quinn KP, Rajaram N, Balachandran K (2016). Valve interstitial cell contractile strength and metabolic state are dependent on its shape. Integr Biol (Camb).

[20] Vargas I (2018). Rapid quantification of mitochondrial fractal dimension in individual cells. Biomed Opt Express.

[21] Rice WL, Kaplan DL, Georgakoudi I (2010). Two-Photon Microscopy for Non-Invasive, Quantitative Monitoring of Stem Cell Differentiation. Plos One.

[22] Meleshina AV (2017). Two-photon FLIM of NAD(P)H and FAD in mesenchymal stem cells undergoing either osteogenic or chondrogenic differentiation. Stem Cell Res Ther.

[23] Baugh LM (2017). Non-destructive two-photon excited fluorescence imaging identifies early nodules in calcific aortic-valve disease. Nature biomedical engineering.

[24] Lam NT, Tandon I, Balachandran K (2019). The role of fibroblast growth factor 1 and 2 on the pathological behavior of valve interstitial cells in a three-dimensional mechanically-conditioned model. Journal of Biological Engineering.

[25] Rutkovskiy A (2017). Valve Interstitial Cells: The Key to Understanding the Pathophysiology of Heart Valve Calcification. Journal of the American Heart Association.

[26] Yip, C. Y., Chen, J. H., Zhao, R. & Simmons, C. A. Calcification by Valve Interstitial Cells Is Regulated by the Stiffness of the Extracellular Matrix. *Arterioscler Thromb Vasc Biol*, 10.1161/ATVBAHA.108.182394 (2009). 10.1161/ATVBAHA.108.182394 19304575

[27] Yu P-J (2009). Correlation between plasma osteopontin levels and aortic valve calcification: Potential insights into the pathogenesis of aortic valve calcification and stenosis. The Journal of Thoracic and Cardiovascular Surgery.

[28] Yang X (2009). Bone morphogenic protein 2 induces Runx2 and osteopontin expression in human aortic valve interstitial cells: Role of Smad1 and extracellular signal-regulated kinase 1/2. The Journal of Thoracic and Cardiovascular Surgery.

[29] Towler DA (2013). Molecular and cellular aspects of calcific aortic valve disease. Circ Res.

[30] Gu X, Masters KS (2011). Role of the Rho pathway in regulating valvular interstitial cell phenotype and nodule formation. American journal of physiology. Heart and circulatory physiology.

[31] Atkinson L (2018). Reversal of stress fibre formation by Nitric Oxide mediated RhoA inhibition leads to reduction in the height of preformed thrombi. Sci Rep.

[32] Gould ST, Matherly EE, Smith JN, Heistad DD, Anseth KS (2014). The role of valvular endothelial cell paracrine signaling and matrix elasticity on valvular interstitial cell activation. Biomaterials.

[33] Tandon I (2016). Valve interstitial cell shape modulates cell contractility independent of cell phenotype. Journal of Biomechanics.

[34] Bowler MA, Merryman WD (2015). *In vitro* models of aortic valve calcification: solidifying a system. Cardiovasc Pathol.

[35] Butcher JT, Nerem RM (2006). Valvular endothelial cells regulate the phenotype of interstitial cells in co-culture: effects of steady shear stress. Tissue Eng.

[36] Latif N (2015). Modulation of Human Valve Interstitial Cell Phenotype and Function Using a Fibroblast Growth Factor 2 Formulation. PLoS One.

[37] Miller JD, Weiss RM, Heistad DD (2011). Calcific Aortic Valve Stenosis: Methods, Models, and Mechanisms. Circ Res.

[38] Monzack EL, Masters KS (2011). Can valvular interstitial cells become true osteoblasts? A side-by-side comparison. The Journal of heart valve disease.

[39] Porras AM (2017). Robust Generation of Quiescent Porcine Valvular Interstitial Cell Cultures. J Am Heart Assoc.

[40] Monzack EL, Masters KS (2012). A time course investigation of the statin paradox among valvular interstitial cell phenotypes. American journal of physiology. Heart and circulatory physiology.

[41] Grau JB (2012). Analysis of osteopontin levels for the identification of asymptomatic patients with calcific aortic valve disease. The Annals of thoracic surgery.

[42] Farrar EJ, Pramil V, Richards JM, Mosher CZ, Butcher JT (2016). Valve interstitial cell tensional homeostasis directs calcification and extracellular matrix remodeling processes via RhoA signaling. Biomaterials.

[43] Lam AY, Mirzaei Z, Wei K, Simmons CA (2018). FHL2-RhoA Signalling Regulates Mechanically Induced Aortic Valve Interstitial Cell Osteogenic Differentiation. Atherosclerosis Supplements.

[44] Ong S-B, Hausenloy DJ (2010). Mitochondrial morphology and cardiovascular disease. Cardiovascular Research.

[45] Gregory CA, Gunn WG, Peister A, Prockop DJ (2004). An Alizarin red-based assay of mineralization by adherent cells in culture: comparison with cetylpyridinium chloride extraction. Anal Biochem.

[46] Jones JD, Ramser HE, Woessner AE, Quinn KP (2018). *In vivo* multiphoton microscopy detects longitudinal metabolic changes associated with delayed skin wound healing. Communications Biology.

[47] Duan B, Hockaday LA, Kapetanovic E, Kang KH, Butcher JT (2013). Stiffness and adhesivity control aortic valve interstitial cell behavior within hyaluronic acid based hydrogels. Acta Biomater.

