# Supplementary material for: Label-free metabolic biomarkers for assessing valve interstitial cell calcific progression
Source: Sci Rep. 2020 Jun 25;10:10317. doi: 10.1038/s41598-020-66960-4 (PMC7316720; doi:10.1038/s41598-020-66960-4)
Supplement: Supplementary file 1 — Supplementary Information. [file 41598_2020_66960_MOESM1_ESM.docx]

Supplementary Information

Label-free metabolic biomarkers for assessing valve interstitial cell calcific progression

Ishita Tandon^#,1^, Olivia I. Kolenc^#,1^, Delaney Cross^1^, Isaac Vargas^1^, Shelby Johns^1^,

*Kyle P. Quinn^1^, *Kartik Balachandran^1^

^1^Department of Biomedical Engineering, University of Arkansas, Fayetteville AR 72701 USA

*Address all correspondence to:

| Kyle P. Quinn, PhD  Department of Biomedical Engineering  University of Arkansas  123 John A. White Jr. Engineering Hall  Fayetteville AR 72701  Tel: +1 479 575 5364  Fax: +1 479 575 4346  E-mail: kpquinn@uark.edu | Kartik Balachandran, Ph.D.  Department of Biomedical Engineering  University of Arkansas  122 John A. White Jr. Engineering Hall  Fayetteville AR 72701  Tel: +1 479 575 3376  Fax: +1 479 575 4346  E-mail: kbalacha@uark.edu |
| --- | --- |

# Both authors contributed equally to the manuscript.

METHODS AND RESULTS

Preparation of stiff and compliant substrates for modeling calcification progression in valve interstitial cells

|  | Sylgard 527 A:B in 1:1 ratio | | Sylgard 184 Base:Curing in 10:1 ratio | |  |
| --- | --- | --- | --- | --- | --- |
|  | A | B | Base | Curing Agent | Total |
| \|  \| \| --- \| | Amount (%) | | | |  |
| Compliant substrate | 49.4 | 49.4 | 1.1 | 0.1 | 100 |
| Stiff substrate | 41.8 | 41.8 | 14.9 | 1.5 | 100 |

Substrates were created by spin coating[^1^](#_ENREF_1) 25 mm no. 1 coverslips with polydimethylsiloxane (PDMS) Sylgard 184 (Dow Corning, Midland, MI) and Sylgard 527 (Dow Corning) in varying ratios (Supplementary Table S1) to obtain compliant or stiff substrates[^2^](#_ENREF_2). The tangent modulus of compliant and stiff substrates was measured using standard uniaxial mechanical testing protocols from five different samples prepared on two different days[^2^](#_ENREF_2). Briefly, 2 mm thick tensile strips were cut and uniaxially stretched at 2mm/min, until failure using Instron 5900 Series (Canton, MA). Sample tangent moduli of these samples was quantified from the linear region of the stress strain curves (Supplementary Fig. S1). The compliant matrices had a modulus of 4.98 ± 0.51 kPa and the stiffer matrices had a modulus of 88.32 ± 10.06 kPa.

Supplementary Table S1. Composition of the PDMS substrate

Gene expression profile for structural and phenotypic markers of CAVD progression

QRT-PCR was performed to assess the gene expression of ACTA2 (Supplementary Fig. S2a), a marker for myofibroblastic activation, RHOA (Supplementary Fig. 2b), a regulator of calcific nodule formation[^3^](#_ENREF_3), TGFβR1 (Supplementary Fig. S2c), a receptor for the TGFβ1, 2 and 3 ligands, known for maintaining valve integrity[^4^](#_ENREF_4), OPN (Supplementary Fig. S2d), an early marker for CAVD progression[^5^](#_ENREF_5), OCN (Supplementary Fig. S2e), a late marker for CAVD progression[^6^](#_ENREF_6), and RUNX2 (Supplementary Fig. S2f), a key transcription factor that regulates osteogenic differentiation[^7^](#_ENREF_7). Expression was quantified as fold change (2^-ΔΔCt^) with respect to the housekeeping gene 18S, and day 1 ΔCt average values. Gene expression did not show any statistically significant differences between groups.

Redox field of view-field of view (FOV-FOV) variability

The FOV-FOV variability in the ORR and FD metrics were calculated as the average of the standard deviation among the FOVs in a sample per treatment condition per time point. The FOV-FOV variability in ORR ranged around 5% of the average ORR values (Supplementary Fig. S3a) and 2 % for FD (Supplementary Fig. S3b).


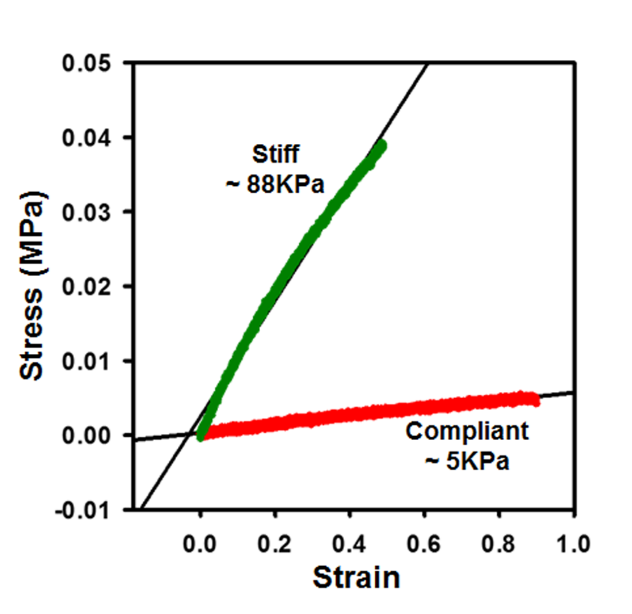


Supplementary Figure S1. Representative stress-strain graph from uniaxial mechanical test of stiff and compliant substrate used in our study.

Supplementary Figure S2. Gene expression profiles of structural and phenotypic markers during VIC osteogenesis.


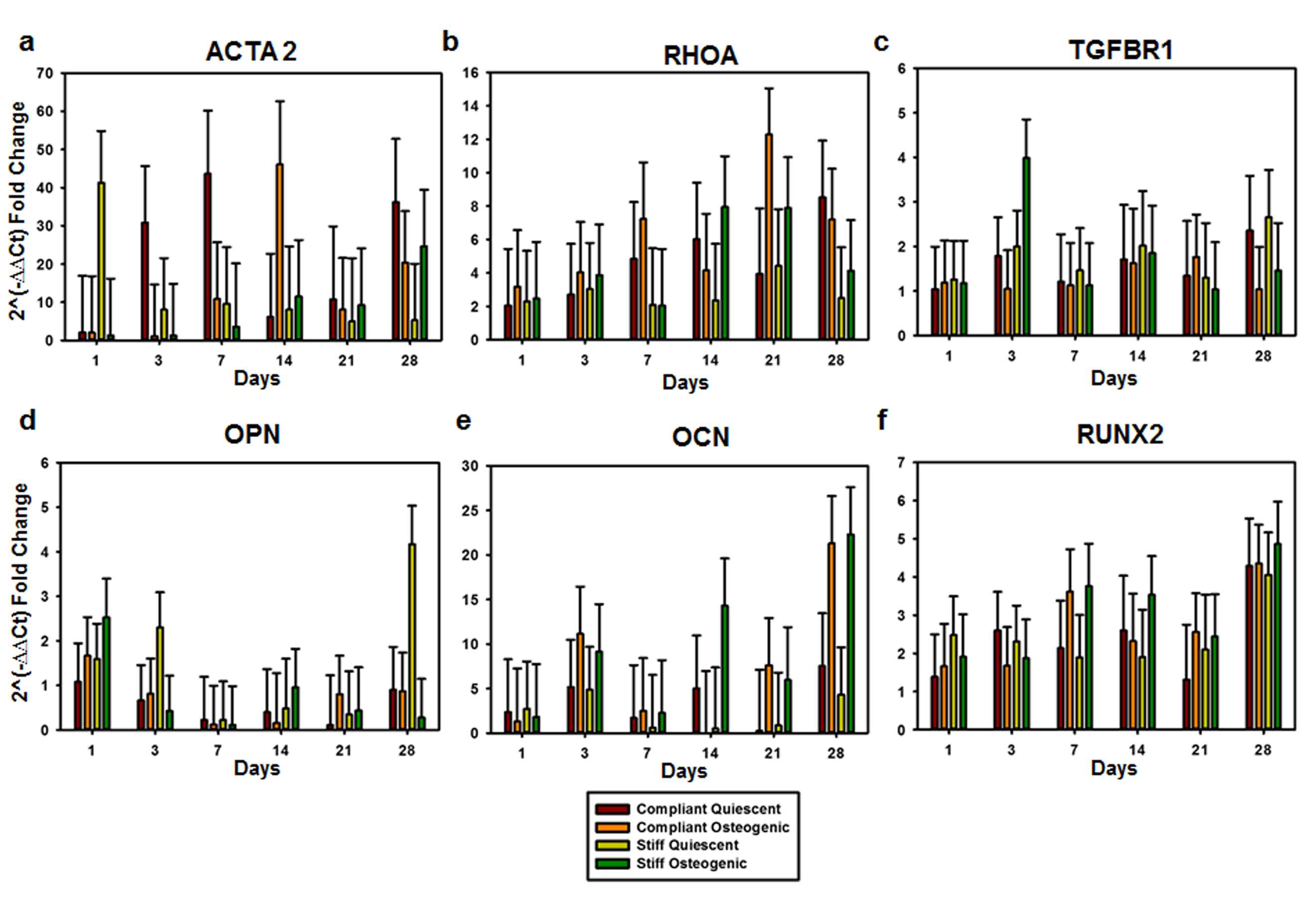


Fold change for (a) ACTA2, (b) RHOA, (c) TGFβR1, (d) OPN, (e) OCN and (f) RUNX2 gene expression for VIC 2D cultures for days 1-28 under quiescent or osteogenic conditions on compliant or stiff substrates. N=3-7.

Supplementary Figure S3. Variability in fields-of-view (FOV-FOV) for Optical redox ratio and Fractal dimension


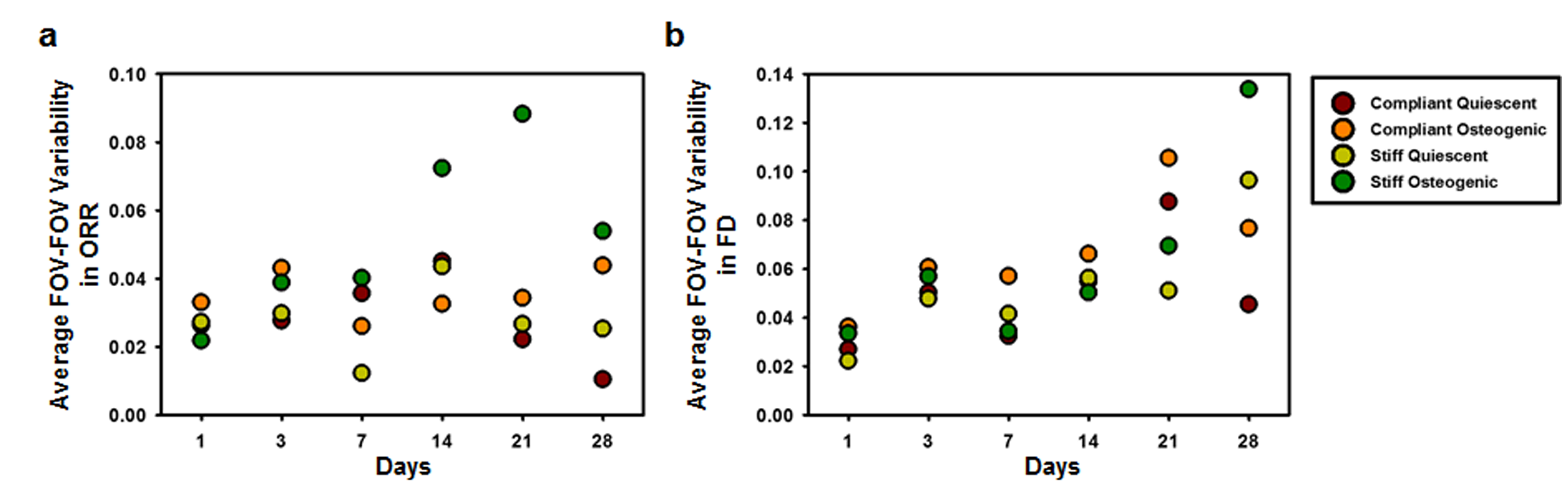


Standard deviation among the FOVs in a sample per treatment condition per time point in (a) Optical redox ratio (ORR) and (b) Fractal dimension (FD).


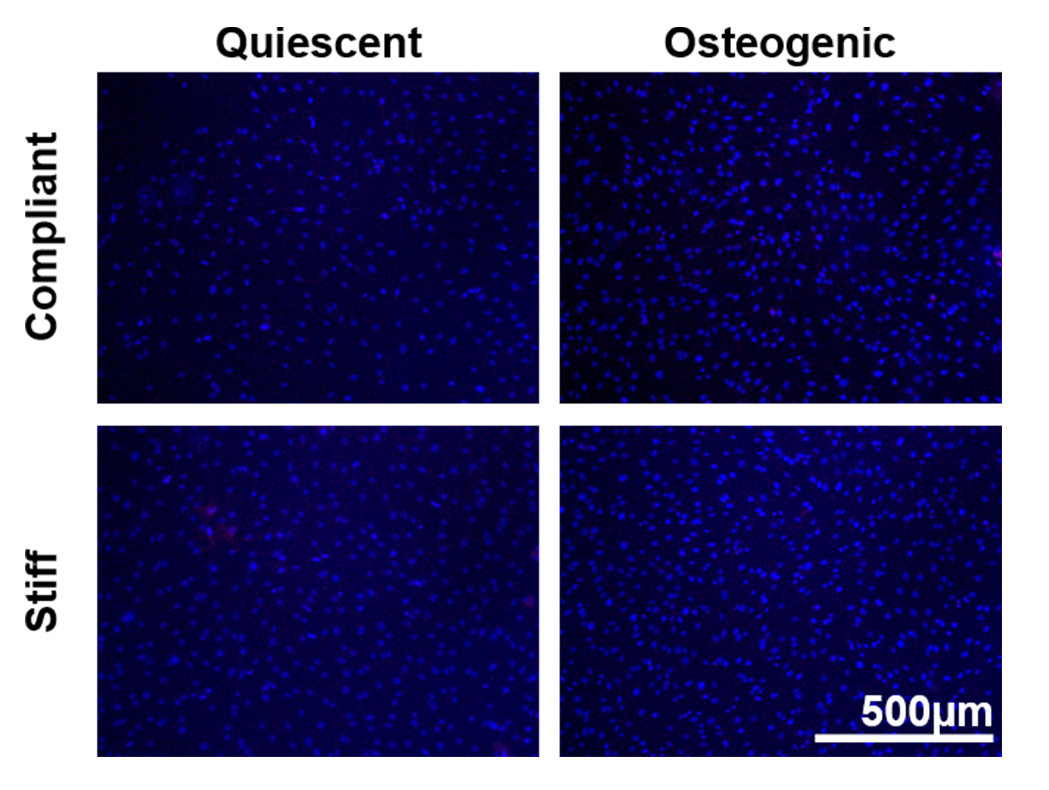


Supplementary Figure S4. No primary negative controls for proliferation and apoptosis analysis for stiff and compliant substrates in quiescent and osteogenic cultures.

REFERENCES

1 Tandon, I. *et al.* Valve interstitial cell shape modulates cell contractility independent of cell phenotype. *Journal of biomechanics* 49, 3289-3297 (2016).

2 Palchesko, R. N., Zhang, L., Sun, Y. & Feinberg, A. W. Development of Polydimethylsiloxane Substrates with Tunable Elastic Modulus to Study Cell Mechanobiology in Muscle and Nerve. *PLOS ONE* 7, e51499, doi:10.1371/journal.pone.0051499 (2012).

3 Gu, X. & Masters, K. S. Role of the Rho pathway in regulating valvular interstitial cell phenotype and nodule formation. *American journal of physiology. Heart and circulatory physiology* 300, H448-H458, doi:10.1152/ajpheart.01178.2009 (2011).

4 Towler, D. A. Molecular and cellular aspects of calcific aortic valve disease. *Circ Res* 113, 198-208, doi:10.1161/CIRCRESAHA.113.300155 (2013).

5 Grau, J. B. *et al.* Analysis of osteopontin levels for the identification of asymptomatic patients with calcific aortic valve disease. *The Annals of thoracic surgery* 93, 79-86, doi:10.1016/j.athoracsur.2011.08.036 (2012).

6 Osman, L., Yacoub, M. H., Latif, N., Amrani, M. & Chester, A. H. Role of Human Valve Interstitial Cells in Valve Calcification and Their Response to Atorvastatin. *Circulation* 114, I-547-I-552, doi:doi:10.1161/CIRCULATIONAHA.105.001115 (2006).

7 Rutkovskiy, A. *et al.* Valve Interstitial Cells: The Key to Understanding the Pathophysiology of Heart Valve Calcification. *Journal of the American Heart Association* 6, e006339, doi:10.1161/JAHA.117.006339 (2017).
